# RSV Immunoprophylaxis in Infants and Children: Old Standards, New Agents and the Complexities Therein

**DOI:** 10.3390/vaccines14070556

**Published:** 2026-06-25

**Authors:** Bosco A. Paes, Paolo Manzoni, John R. Fullarton, Barry S. Rodgers-Gray, Xavier Carbonell-Estrany

**Affiliations:** 1 Hamilton Health Sciences, McMaster University, Hamilton, ON L8N 3Z5, Canada; 2 Department of Public Health and Pediatric Sciences, University of Torino School of Medicine, 10124 Turin, Italy; 3 Division of Paediatrics and Neonatology, Degli Infermi Hospital, 13875 Ponderano, Italy; 4 Violicom Medical Limited, Aldermaston RG7 8AP, UKbarry@violicom.co.uk (B.S.R.-G.); 5 Department of Neonatology, Hospital Clinic, 08028 Barcelona, Spain

**Keywords:** respiratory syncytial virus, RSV, monoclonal antibody, palivizumab, nirsevimab, clesrovimab, immunoprophylaxis, infant

## Abstract

Every year, respiratory syncytial virus (RSV) causes an estimated 33 million lower respiratory tract infections in children under five years of age, driving millions of hospitalizations worldwide and substantial mortality in developing countries. For 28 years, the monoclonal antibody (mAb) palivizumab has been the principal agent for RSV immunoprophylaxis, reducing hospitalization in defined high-risk groups through monthly intramuscular dosing. The recent approval of two second-generation long-acting mAbs, nirsevimab and clesrovimab, and maternal preF vaccine has fundamentally changed the RSV prevention landscape. In contrast to palivizumab, the long-acting mAbs offer single-dose seasonal protection across a broader infant population, enabling universal immunization programmes for the first time. In this review, we conjointly examine nirsevimab and clesrovimab across their mechanisms of action, pharmacokinetics, efficacy, safety and cost-effectiveness, using palivizumab as the reference standard. Cross-trial efficacy comparisons are complicated by differences in study populations and endpoint definitions; however, when these factors are considered, the available evidence suggests that all three agents offer broadly comparable protection against severe RSV disease. All three agents also demonstrate favourable and comparable tolerability profiles. Nirsevimab is now supported by a substantial body of real-world evidence confirming effectiveness in routine immunization programmes that closely align with registrational studies. Clesrovimab, as the newest agent, currently lacks real-world effectiveness, and both long-acting monoclonals require further confirmatory evidence in high-risk groups. Overall, existing data support that both monoclonals have equivalent efficacy and safety profiles as palivizumab, and choice should be based on cost-effectiveness and local availability, with consideration given to optimal integration of infant immunoprophylaxis alongside maternal RSV vaccination programmes.

## 1. Introduction

Respiratory syncytial virus (RSV) is the primary cause of lower respiratory tract infections (LRTIs) in infants and young children worldwide, responsible for an estimated 33 million episodes, 3.6 million hospital admissions and 26,300 in-hospital deaths annually in children under five years of age [1,2]. It is well-established that the greatest morbidity and mortality burden falls within the first six months of life, and more predominantly in children less than two months of age [1,3]. While RSV causes significant illness across early childhood, preterm infants are particularly vulnerable due to reduced maternal antibody transfer, immature lung development and impaired innate immunity, with the risk of severe illness inversely correlated with gestational age (GA) at birth [4,5]. In addition, infants with chronic lung disease (CLD)/bronchopulmonary dysplasia (BPD), hemodynamically significant congenital heart disease (CHD), Down syndrome, and those with compromised immunity are at increased risk of severe outcomes [6,7,8,9,10,11].

For more than 25 years, palivizumab, a humanized monoclonal antibody (mAb) targeting the RSV fusion (F) protein, has been the principal licensed agent used for RSV immunoprophylaxis [2,12]. Demonstrated to reduce RSV-associated hospitalization in high-risk infant populations (preterm ≤ 35 weeks’ GA [wGA], CLD/BPD and CHD), palivizumab became the established standard of care [2,13,14]. However, its use has been constrained by the need for monthly intramuscular dosing across the RSV season, its high cost and recommendations restricting prophylaxis to subpopulations of high-risk infants and children [13,15,16,17,18].

Advances in the structural understanding of RSV F, particularly the prefusion conformation, together with antibody engineering, have enabled the development of long-acting mAbs with greater neutralization potency than palivizumab [15,17]. These agents incorporate modifications that can extend serum half-life, enabling single-dose seasonal protection and, for the first time, the ability to deliver universal infant immunoprophylaxis [13,15,17,19]. The long-acting mAbs nirsevimab and clesrovimab are both approved for infants entering their first RSV season across the gestational age spectrum [20,21], representing a fundamental shift from the targeted, multi-dose paradigm of palivizumab [17]. Nirsevimab is also licensed for the protection of high-risk children entering their second RSV season [20], with similar licensure expected for clesrovimab in due course. The RSV prevention landscape has been further expanded by maternal RSV vaccination (RSVpreF), which confers neonatal protection via transplacental antibody transfer and is now recommended in several countries as a complementary strategy for protecting young infants [18,22,23,24]. A range of novel vaccines, next-generation mAbs, and combination immunoprophylactic strategies are also under active clinical investigation [13,25].

The introduction of long-acting mAbs and RSVpreF has prompted major national and international bodies, including the World Health Organization, the Canadian National Advisory Committee on Immunization, the American Academy of Pediatrics and the Advisory Committee on Immunization Practices to update their guidance [24,26,27,28], and multiple countries across the world, including France, Spain, Chile, Canada and the United States have now implemented broad infant nirsevimab immunization programmes [18]. As health systems transition from targeted palivizumab prophylaxis to broader long-acting mAb strategies, clinicians and policymakers require clear, evidence-based comparisons to guide agent selection and programme design. No randomized head-to-head trial has directly compared nirsevimab versus clesrovimab, and indirect cross-trial comparisons are complicated by heterogeneity in study populations, endpoint definitions and RSV season characteristics [14,29,30].

This narrative review provides a structured, comparative analysis of nirsevimab and clesrovimab for RSV prevention in children, using palivizumab as the reference standard. It examines their product characteristics, mechanisms of action, pharmacokinetic properties, potency, clinical efficacy and effectiveness, safety profiles, effects on longer-term respiratory outcomes, cost-effectiveness and current recommendations for use. By bringing together the available evidence, this review aims to support clinicians, immunization programme leads, health agencies and technology assessment bodies as they make decisions within a rapidly changing RSV prevention landscape.

## 2. Product Profiles

The three agents discussed in this review are all passive immunoprophylactic monoclonal antibodies used for RSV prevention and administered by intramuscular injection [13,17]. Unlike vaccines, they provide passive antibody-mediated protection rather than inducing the recipient’s own active immune response [18,31,32,33].

Palivizumab is indicated for the prevention of serious RSV-related lower respiratory tract disease in high-risk infants, including those born prematurely (≤35 weeks’ gestational age) and those aged under two years with BPD or hemodynamically significant CHD. It is administered as monthly 15 mg/kg injections during the RSV season [12].

Nirsevimab is approved for a broader population encompassing all infants entering or during their first RSV season, as well as high-risk children up to 24 months of age entering their second season. A single weight-banded dose (50 mg for infants <5 kg; 100 mg for ≥5 kg) provides first season-long protection [13,20].

Clesrovimab received FDA approval in June 2025 and European Medicines Agency (EMA) approval in April 2026 for the prevention of RSV lower respiratory tract disease in neonates and infants entering or during their first RSV season. It is administered as a single 105 mg dose, with no adjustment for infant weight [21,34].

### 2.1. Site of Action

RSV infection is initiated by the F protein of the virus, which mediates its entry into host cells by driving membrane fusion between the virus and the target cell [17,34,35]. The F protein exists in two distinct conformational states: a metastable prefusion form and a stable postfusion form [17,35,36]. The prefusion conformation is the primary target for neutralizing antibodies, as it exposes highly conserved antigenic sites that are either absent or poorly accessible once the protein transitions to the postfusion state [17,35].

Palivizumab binds to antigenic site II (also referred to as site A), a region present on both the prefusion and postfusion conformations of the F protein [17,35]. Whilst site II is accessible and well characterized, antibodies targeting prefusion-specific epitopes are considerably more potent neutralizers than those binding shared sites such as site II [35]. Indeed, nirsevimab targets antigenic site Ø, a highly conserved epitope exclusive to the prefusion F protein and considered among the most potently neutralizing on the RSV F protein [17,35]. Clesrovimab binds to antigenic site IV, a region present on both the prefusion and postfusion conformations of the F protein, representing a structurally and immunologically distinct target from both palivizumab and nirsevimab. Site IV is associated with high neutralization potency in preclinical and early clinical evaluations [17,37,38].

The availability of mAbs targeting distinct epitopes on the RSV F protein may itself help to mitigate the risk of viral resistance, as no single mutation is likely to confer cross-resistance across binding sites [38]. In practice, clinically significant resistance remains rare for all three agents in circulating strains [39]. For palivizumab, despite case reports documenting escape mutations following prophylaxis exposure [40,41], there are no meaningful data demonstrating that mutational resistance has emerged at a population level [13]. For nirsevimab, a French multicentre observational study conducted during the 2023–24 RSV season found no resistance-associated substitutions in RSV-A breakthrough infections, although resistant RSV-B variants were identified in 2 of 24 cases [42]. A subsequent study by the same investigators, conducted during the 2024–25 season when RSV-B predominated, identified resistance-associated substitutions in approximately 12.5% of RSV-B breakthrough infections, with greater mutational diversity than previously observed [43]. Together, these findings highlight the importance of ongoing molecular surveillance to monitor the emergence and spread of resistant variants. The authors also raised the question of whether more systematic virological characterization, including seasonal RSV genotyping, may be needed as nirsevimab use increases globally. For clesrovimab, published resistance data remain very limited; however, an analysis of over 15,000 RSV A and B sequences in GenBank showed that the site IV binding epitope was conserved with 99.8% identity, suggesting a similarly low risk of clinically meaningful resistance [38].

### 2.2. Half-Life and Potency

A pharmacological limitation of palivizumab is its unmodified IgG1 Fc region, which confers a serum half-life of approximately 20 days in infants. As a result, monthly intramuscular dosing is required to maintain protective concentrations throughout a typical 5-month RSV season [12,44]. By contrast, nirsevimab incorporates a triple amino acid YTE substitution (Met252Tyr/Ser254Thr/Thr256Glu) in the Fc region, which enhances binding to the neonatal Fc receptor expressed predominantly on endothelial cells, reducing lysosomal degradation and resulting in a prolonged serum half-life. In infants, its serum half-life is approximately 71 days, allowing a single intramuscular dose to maintain protective neutralizing antibody levels across a typical RSV season [15,20,45]. Clesrovimab also has the YTE triple amino acid substitution in the Fc region. Its half-life is shorter than nirsevimab at approximately 44 days, but it is still efficacious for single-dose seasonal prophylaxis [21,37,38].

In terms of neutralization potency, evidence increasingly suggests that both nirsevimab and clesrovimab have greater RSV-neutralizing activity than palivizumab. Notably, nirsevimab has been shown to produce approximately 10-fold higher and more sustained neutralizing antibody levels in vivo compared with palivizumab [15,17].

A summary of the comparative profiles of palivizumab, nirsevimab and clesrovimab can be found in Table 1.

## 3. Efficacy

### 3.1. Pivotal Clinical Trial Data

The clinical efficacy of palivizumab, nirsevimab and clesrovimab has been evaluated across a series of randomized controlled trials, though direct comparison across agents is complicated by important differences in study populations, endpoint definitions and the RSV seasons in which trials were conducted. A summary of pivotal clinical trial data is presented in Table 2.

Palivizumab was the first mAb shown to be efficacious in preventing RSV in the landmark IMpact-RSV trial, a multicentre, randomized, double blind, placebo-controlled study published in 1998 [46]. Among preterm infants born at 35 weeks’ gestational age or earlier, palivizumab reduced RSV-related hospitalization rates by 78% (95% CI 66–90; *p* < 0.001) compared with placebo. However, when the entire trial population was analyzed, including infants with CLD/BPD, the overall reduction in hospitalization was 55% [46]. This latter figure is sometimes quoted as the overall efficacy of palivizumab and then negatively compared with the prevention of ~80% for nirsevimab and clesrovimab. However, the 39% reduction observed in the CLD/BPD subgroup (95% CI 20–58; *p* = 0.038) reflects the greater disease burden and immunological complexity within this group rather than reduced effectiveness of palivizumab itself [7]. It is worth noting that the CLD/BPD population was not included in the registrational trials of nirsevimab or clesrovimab [38,47,48].

Palivizumab efficacy has also been demonstrated in infants with hemodynamically significant CHD, where a dedicated randomized trial reported a significant reduction in RSV-related hospitalization compared with a placebo [49]. Real-world data are consistent with trial findings; the Palivizumab Outcomes Registry, which included 19,548 infants receiving at least one dose, recorded an RSV hospitalization rate of just 1.3% in treated infants [50], and a systematic review of six real-world cohort studies in moderate-to-late preterm infants reported an approximate four-fold reduction in RSV hospitalization compared with untreated infants [16,51].

Nirsevimab has been assessed in two pivotal randomized controlled trials [47,48]. One study focused on preterm infants born at 29–35 weeks’ gestational age and found that nirsevimab reduced medically attended RSV-associated lower respiratory tract disease (LRTD), the primary endpoint, by 70.1% compared with placebo (95% CI 52.3–81.2; *p* < 0.001). RSV-related hospitalization was reduced by 78.4% (95% CI 51.9–90.3; *p* < 0.001) [47]. The subsequent MELODY trial evaluated nirsevimab in a broader population of infants born at 35 weeks’ gestational age or later. In this cohort, medically attended RSV-associated LRTD was reduced by 74.5% (95% CI 49.6–87.1; *p* < 0.001). RSV-related hospitalization was reduced by 62.1% (95% CI −8.6–86.8; *p* = 0.07), although this did not reach statistical significance [48]. The HARMONIE trial provided further supportive evidence in a real-world-like setting, demonstrating a significant reduction in RSV-related hospitalization among all infants up to one year of age [52].

Growing real-world evidence from nirsevimab immunization programmes is consistent with, and in some instances has shown greater efficacy than that observed in clinical trials [16,53,54,55]. For example, in a retrospective cohort of over 400,000 infants in the United States, nirsevimab was associated with a significantly lower risk of RSV-related hospitalization (adjusted hazard ratio 0.23, 95% CI 0.21–0.26) and an approximate 50% reduction in RSV-related intensive care unit admissions compared with untreated infants [16,56].

Clesrovimab was evaluated in the pivotal CLEVER trial, a randomized, double-blind, placebo-controlled study in infants born at 29 weeks’ gestational age or later entering their first RSV season [38]. Clesrovimab reduced medically attended RSV-associated LRTD by 60.4% (95% CI 44.1–71.9; *p* < 0.001) and RSV-related hospitalization by 84.2% (95% CI 66.6–92.6; *p* < 0.001) compared with the placebo [38]. Additional efficacy data are available from the SMART trial, a Phase 3 multicentre, multinational study that compared clesrovimab directly with palivizumab in infants meeting criteria for palivizumab administration, including infants with BPD and CHD, making it the only trial to date to include an active comparator arm across this agent class [37]. Although full results have not yet been published, preliminary data indicate that RSV-associated medically attended LRTD occurred in 3.6% of clesrovimab-treated infants compared with 3.0% of those receiving palivizumab, with RSV-related hospitalization rates of 1.3% and 1.5%, respectively, suggesting broadly comparable efficacy between the two agents in this high-risk population [37]. Real-world evidence for clesrovimab is not yet available, given its recent regulatory approval, and post-implementation data will be important in confirming trial findings across broader and more diverse populations.

### 3.2. Challenges in Cross-Trial Interpretation

Interpretation of efficacy data across these three agents is complicated by the absence of a universally standardized definition for RSV-associated LRTD or LRTI. The IMpact-RSV trial used RSV hospitalization as its primary endpoint [46], whereas the nirsevimab and clesrovimab infant trials used medically attended RSV-associated lower respiratory tract disease or infection (LRTD/LRTI) endpoints, which incorporate both RSV-related hospitalization and medical attendance, and were defined differently across studies [38,47,48]. These differences indicate that caution should be used when comparing the headline efficacy figures across the respective trials, and, indeed, that any variation in results may reflect differences in endpoint sensitivity and baseline population risk as much as genuine differences in drug performance.

### 3.3. Indirect Comparisons and Network Meta-Analyses

In the absence of head-to-head trial data, indirect comparisons and network meta-analyses have sought to contextualize the relative efficacy of these agents. A meta-analysis by Fullarton et al. examined the efficacy of palivizumab in preventing medically attended RSV infections (MARI) in otherwise healthy preterm infants, using an endpoint that was used in the nirsevimab trials. The efficacy estimates reported found palivizumab and nirsevimab to have similar efficacy at preventing MARI [57]. A systematic review by Vaduva et al. concluded that nirsevimab demonstrates comparable or superior efficacy to palivizumab in preventing RSV LRTIs when populations are appropriately considered [16]. Another systematic review and network meta-analysis by Nosho et al., which included palivizumab, nirsevimab and clesrovimab, found that the three agents have broadly similar efficacy when comparable populations and endpoints are assessed [14]. Overall, the available indirect evidence supports the conclusion that all three agents offer meaningful and broadly equivalent protection against serious RSV disease in appropriately selected populations, and that treatment decisions should be guided by population eligibility, dosing practicality, availability and cost-effectiveness.

## 4. Long-Term Respiratory Morbidity

RSV LRTI in early infancy has been epidemiologically and mechanistically linked to recurrent wheezing and asthma in later childhood [58,59]. However, it remains debated whether RSV infection directly causes asthma or preferentially affects predisposed infants [58,59,60]. As early RSV LRTI contributes to subsequent wheezing and asthma, effective prophylaxis in infancy can reduce longer-term airway disease burden [60]. The WOO-353 Reactive Airway Disease study, a prospective study of preterm infants ≤ 35 wGA, found that palivizumab prophylaxis was associated with an approximate 80% reduction in the relative risk of recurrent wheezing between 2 and 5 years of age among children without an atopic background [61]. However, this protective effect was not observed in children with an atopic background, suggesting that an atopic disposition obviated any protection afforded by a reduction in severity of RSV disease [61]. The MAKI trial, a prospective, randomized, placebo-controlled study in healthy preterm infants born at 32–35 weeks’ gestational age, reported a reduced risk of parent-reported infrequent wheezing at age 6 years in those receiving palivizumab. However, palivizumab prophylaxis did not have a significant effect on asthma or lung function at 6 years of age [62]. The SCELIA study similarly found that palivizumab prophylaxis in preterm infants was associated with a lower incidence of recurrent wheezing during the first 6 years of life, although it did not prevent the onset of atopic asthma [63].

For nirsevimab, analysis by the TriNetX Research Network found that prophylaxis was associated with a significantly lower subsequent risk of wheezing for up to 1 year in real-world practice [64]. These findings provide early evidence that longer-term respiratory benefits may extend to newer long-acting mAbs, which may be expected, since the reduction in subsequent wheezing and asthma is a direct result of reducing severe RSV disease. No equivalent data are yet available for clesrovimab, and post-marketing studies capturing these endpoints will be important in confirming that similar benefits are observed.

## 5. Safety

The safety of palivizumab, nirsevimab and clesrovimab has been evaluated throughout their clinical development programmes. A comparison of their safety findings is summarized in Table 3.

### 5.1. Adverse Event Profiles from Pivotal Trials

In the IMpact-RSV trial, palivizumab monthly injections were well tolerated [46]. The most commonly reported adverse events were fever, nervousness and injection site reaction, with rates comparable between palivizumab and placebo groups. Discontinuation of palivizumab injections due to adverse events was rare, occurring in only 0.3% of patients [46]. The pivotal nirsevimab trials also demonstrated a similar adverse event profile. The overall incidence of adverse events and serious adverse events was comparable between nirsevimab and placebo arms [47,48]. In the HARMONIE trial, no substantial safety signals were identified [52]. In the CLEVER trial, the proportion of infants experiencing adverse events was generally similar between the clesrovimab and placebo groups. Injection-site pain was the most commonly reported injection-site adverse event, while irritability was the most common systemic adverse event [38].

### 5.2. Post-Marketing Safety Data

Palivizumab has an extensive post-marketing safety record accumulated over more than 25 years of clinical use across millions of doses worldwide [2]. Post-marketing surveillance has not identified any new or unexpected safety signals beyond those characterized in the pivotal trials, and the overall safety profile has remained consistent across diverse real-world populations and settings [2,16].

Post-marketing safety data for nirsevimab are accumulating rapidly following its broad implementation in universal immunization programmes across several countries [16]. The tolerability profile in real-world practice appears consistent with trial data [16]. For clesrovimab, post-marketing safety data are not yet available, given its recent regulatory approval.

### 5.3. Immunogenicity

Anti-drug antibody (ADA) development has been assessed for all three agents. For palivizumab, ADA rates have been reported as low and without apparent clinical consequence [2,65]. Nirsevimab demonstrated low immunogenicity in clinical trials, with ADA development observed in a small proportion of participants and no association with altered pharmacokinetics or reduced clinical efficacy reported [47,48]. For clesrovimab, ADA rates were similarly low in the CLEVER trial, with no clinically meaningful impact on drug exposure or efficacy identified [38].

## 6. Cost Effectiveness

Economic evaluations of RSV mAb prophylaxis have evolved considerably with the introduction of longer-acting agents, shifting the analytical focus from targeted strategies for high-risk infants and children to the cost-effectiveness of universal infant immunization programmes.

For palivizumab, economic analyses have reported a wide range of cost-effectiveness estimates, reflecting differences in the populations receiving prophylaxis, country-specific RSV epidemiology, and modelling approaches [2]. Overall, most studies have found palivizumab to be cost-effective in BPD, CHD and <32 wGA preterm populations, whereas in moderate-to-late preterm infants (born ≥ 32–35 wGA) cost-effectiveness has been demonstrated only when additional risk factors, such as presence of siblings, maternal or household smoking and younger chronological age (birth 3 months before to 2 months after the start of the RSV season), have been considered [2]. The challenges of demonstrating cost-effectiveness for palivizumab are largely driven by the high acquisition cost of palivizumab, the need for repeated monthly dosing, and the lower rate of RSV-related hospitalizations in moderate-to-late preterm infants versus their counterparts with BPD or CHD [16].

The single-dose schedules of both nirsevimab and clesrovimab offer meaningful health-economic advantages over palivizumab by reducing administration burden and the need for repeated monthly dosing across the RSV season [16,66]. Economic modelling of nirsevimab in universal immunization programmes across a range of countries has shown it can be a cost-effective option at the right acquisition price [16]. Studies have reported favourable cost-per-hospitalization-averted and cost-per-quality-adjusted life year (QALY) outcomes, particularly when wider reductions in RSV burden at the population level are included [67,68]. The shift from targeted to universal strategies also simplifies programme delivery and reduces the risk of eligible infants being missed, which has indirect cost and equity benefits.

For clesrovimab, early health economic modelling suggests a broadly comparable cost-effectiveness profile to nirsevimab in the United States, with a single-dose regimen similarly supporting favourable cost-per-QALY estimates relative to no prophylaxis [37]. Head-to-head economic comparisons between nirsevimab and clesrovimab are limited at present. A recent analysis by Merck/MSD, the manufacturer of the clesrovimab, found it to be cost-saving compared to nirsevimab [69]. To date, no independent analyses comparing the cost-effectiveness of the two long-acting mAbs have been conducted. Independent, impartial assessments are important for healthcare policy decisions, and we strongly advocate that such analyses be undertaken. Importantly, cost-effectiveness will likely vary by country depending on negotiated pricing, programme infrastructure and baseline RSV hospitalization rates.

For the two long-acting mAbs, the move toward universal immunization programmes reflects a growing recognition that the broader health economic case for RSV prevention extends beyond direct hospitalization costs to include downstream respiratory morbidity, healthcare utilization and quality of life benefits.

## 7. Current Guidance and Practical Recommendations

The introduction of long-acting mAbs has prompted substantial updates to national and international RSV prevention guidance [18,24,27,28]. Palivizumab has a diminishing role in settings where nirsevimab and clesrovimab are available; however, in countries where these long-acting mAbs have not yet been introduced or are not accessible, it continues to play a significant role. Palivizumab is currently available in 93 countries worldwide and remains widely used in many low- and middle-income countries while they await the introduction of newer RSV preventive mAbs [2,13,57].

The Advancing RSV Management and Disease Awareness (ARMADA) expert consensus statement provides practical guidance on the use of long-acting mAbs across diverse clinical settings, addressing eligibility criteria, timing of administration relative to RSV season onset, catch-up dosing for infants born outside the RSV season, and the use of mAbs alongside maternal RSVpreF vaccination [68]. Updated recommendations predicated on the ARMADA consensus now include available data for clesrovimab and palivizumab as a reference and are summarized in Table 4.

Regarding co-administration, current guidance recommends that where a mother has received RSVpreF vaccine ≥ 14 days before delivery, mAb prophylaxis for the infant is generally not required to have one [27,70]. This recommendation reflects the anticipated passive transfer of maternal antibodies, although immunization with a long-acting mAb may still be considered where additional clinical benefit is warranted [70]. In instances where co-administration occurs, interference is considered unlikely [27,68,70]. Anti-viral mAbs such as nirsevimab have a highly specific, virus-directed mechanism of action. As a result, they are not expected to alter the immune response to other immunological products [13,71]. The evidence for immunization with both maternal vaccine and mAb is limited. However, an interim analysis of a recent Phase 4, randomized clinical trial where mother–infant pairs were allocated to either maternal vaccine or nirsevimab alone or combined immunization, reported that at three months follow-up the combined strategy was safe with no related serious adverse events observed in mothers or infants, and yielded high RSV-neutralizing antibody titres in infants [72]. Long-term follow-up findings will help to better define the potential for antibody interactions in this setting.

**Table 4:** Summary of recommendations for RSV prophylaxis with nirsevimab, clesrovimab, and palivizumab in infants and young children [12,20,21,26,27,38,68,70,73,74].

Nirsevimab			Clesrovimab			Palivizumab		
Recommendation	LoE ^a^	GRADE ^b^	Recommendation	LoE ^a^	GRADE ^b^	Recommendation	LoE ^a^	GRADE ^b^
**Term infants without other comorbidities**								
Nirsevimab is recommended for: All infants <8 or <12 months of age at the start of, or born during, their first RSV season	1a	A	Clesrovimab is recommended for: All infants <8 or <12 months of age at the start of, or born during, their first RSV season	1b	A	N/A	-	-
**Preterm infants without other comorbidities**								
Nirsevimab is recommended for: Infants <37 wGA and <8 or <12 months of age at the start of, or born during, their first RSV season ^c^	1a	A	Clesrovimab is recommended for: Infants <37 wGA and <8 or <12 months of age at the start of, or born during, their first RSV season ^c^	1b	A	Palivizumab is recommended for infants: <29 wGA and ≤9 months at the start of the RSV season 29–31 wGA and ≤6 months at the start of the RSV season 32–35 wGA and high-risk using a country-specific or generalisable risk factor scoring tool	1a	A
**Children with CLD/BPD**								
Nirsevimab is recommended for: Children <24 months of age entering their second RSV season with any grade of CLD/BPD	1b	B	Clesrovimab may be considered in: Children <24 months of age with BPD who remain at high-risk for severe RSV through a second RSV season	1b	B	Palivizumab is recommended: For infants ≤12 months at the start of the RSV season During the second year of life, in children who remain at high-risk BPD/CLD, and those at high-risk in the second year of life to be defined according to local experience and practice	1a	A
**Children with HS-CHD**								
Nirsevimab is recommended for: Children <24 months of age entering their second season with uncorrected, palliated cyanotic or acyanotic HS-CHD associated with documented moderate or severe pulmonary hypertension, and/or a requirement for daily medication to manage congestive heart failure or failure to thrive based on CHD status	1b	B	Clesrovimab may be considered in: Children <24 months with HS-CHD entering their second RSV season. Criteria for HS-CHD are similar to those indicated for nirsevimab	1b	B	Palivizumab is recommended for: Infants ≤ 12 months with hemodynamically significant cyanotic or acyanotic disease Children 12–24 months, cyanotic or acyanotic, who remain hemodynamically unstable	1a	A
**Children with other high-risk conditions**								
Nirsevimab is recommended for children <24 months of age entering their second RSV season who have an increased risk for severe RSV. These include: Severe immunosuppression Inborn errors of metabolism Neuromuscular disease Severe pulmonary malformations Genetic syndromes with significant respiratory problems Down syndrome Cystic fibrosis American Indian and Alaska Native heritage; Māori and Pacific ethnicities and individuals in or from First Nations, Inuit, and Métis communities across various settings (e.g., urban, rural, remote, northern)	5 ^d^	D ^d^	Clesrovimab may be considered for children <24 months of age entering their second RSV season who have increased risk for severe RSV. These include: Severe immunosuppression Inborn errors of metabolism Neuromuscular disease Severe pulmonary malformations Genetic syndromes with significant respiratory problems Down syndrome Cystic fibrosis American Indian and Alaska Native heritage; Māori and Pacific ethnicities and individuals in or from First Nations, Inuit, and Métis communities across various settings (e.g., urban, rural, remote, northern)	5 ^d^	D ^d^	Palivizumab is recommended for: Children with Down syndrome ≤ 24 months	2c	B
						Cystic fibrosis (without other comorbidities) ○ Infants ≤ 12 months ○ Children in the second year of life with manifestations of severe lung disease or weigh <10th percentile	2c	C
						Anatomic pulmonary abnormalities or neuromuscular disorder ○ Children ≤24 months with significant neuromuscular disease or congenital anomalies that compromise the respiratory tract (e.g., hypotonia, cerebral palsy, chronic interstitial pulmonary disease, airway and pulmonary malformations, tracheostomy)	4	C
						Immunocompromised children ≤24 months who are profoundly immunocompromised (e.g., primary immunodeficiency syndromes, immune suppression following hematopoietic stem cell transplantation, solid organ transplantation or cytotoxic chemotherapy)	4	C
**Dosing**								
Nirsevimab (weight-based, single dose per season): Weighing < 5 kg: 50 mg (0.5 mL) given IM Weighing ≥ 5 kg: 100 mg (1 mL) given IM Second season only: 200 mg (2 × 100 mg/1 mL) at two IM injection sites Administer soon after birth for infants born during the RSV season, or just prior to RSV season onset for infants born outside the season In endemic countries, a local decision should be made on whether to administer prophylaxis throughout the year or to coincide with peak RSV incidences	1a	A	Clesrovimab (fixed, single dose per season): 105 mg (0.7 mL) given IM, regardless of body weight Administer soon after birth for infants born during the RSV season, or just prior to RSV season onset for infants born outside the season Second-season only: 210 mg (2 × 0.7 mL dose in each thigh)	1b	A	Palivizumab (weight-based, multiple dose per season): 15 mg/kg once a month to cover RSV season (maximum 5 doses *) for all children * Children born during the season will require fewer doses	1a	A

BPD, bronchopulmonary dysplasia; HS-CHD, hemodynamically significant congenital heart disease; CLD, chronic lung disease; IM: intramuscular; RCT, randomized controlled trial; RSV, respiratory syncytial virus; wGA, weeks’ gestational age. ^a^ level of evidence (LoE): 1a = systematic review/meta-analysis of RCTs; 1b = individual RCT; 2a = systematic review of cohort studies; 2b = individual cohort study; 2c = outcomes research/registries; 3a = systematic review of case-control studies; 3b = individual case-control study; 4 = case series; 5 = expert opinion. ^b^ GRADE (Grading of Recommendations Assessment, Development and Evaluation) strength of recommendation: A = consistent level 1 studies; B = consistent level 2 or 3 studies or extrapolations from level 1 studies; C = consistent level 4 studies or extrapolations from level 2 or 3 studies; D = level 5 evidence or troublingly inconsistent or inconclusive studies of any level. ^c^ there are limited clinical data available on the use of nirsevimab and clesrovimab in extremely preterm infants (<29 wGA) who are of chronological age less than 8 weeks. No clinical data are available in infants with a postmenstrual age (GA plus chronological age) of less than 32 weeks [20,21]. ^d^ Supported by 2B to 4C evidence for palivizumab [74].

## 8. Conclusions

Palivizumab, nirsevimab and clesrovimab are all mAb-based RSV immunoprophylactics targeting the RSV F protein, but they differ in epitope specificity, pharmacokinetic profile, approved populations and dosing schedule. Palivizumab represents the first-generation standard used as a comparator mAb for the evaluation of newer agents. In contrast, nirsevimab and clesrovimab offer extended half-lives, single-dose convenience and broader population eligibility, features that have made universal infant immunization programmes a practical reality for the first time.

Despite these differences, the available evidence points to broadly comparable clinical efficacy across all three agents when trial data are interpreted in the right context. The 55% overall risk reduction reported in the IMpact-RSV trial has long shaped perceptions of palivizumab as the least effective option, but this figure encompasses infants with CLD/BPD, a group with greater baseline disease burden in whom a 39% reduction was observed. Among preterm infants without these complicating factors, palivizumab reduced RSV-related hospitalization by 78%, a percentage that sits comfortably alongside the efficacy reported for nirsevimab and clesrovimab in comparable populations. Agent selection should therefore be guided by practical considerations, including approved indication, dosing convenience, programme infrastructure, cost-effectiveness and local availability.

Nirsevimab is now well supported by a substantial and growing body of real-world evidence, while clesrovimab is at an earlier stage with post-implementation data still to emerge. However, important evidence gaps remain true across both agents, particularly around long-term respiratory outcomes, rather than the extrapolated pharmacokinetic bridging data of the effectiveness in special populations, and the interplay between mAb prophylaxis and concurrent maternal RSV vaccination programmes.

Looking ahead, the RSV prevention landscape continues to develop, with next-generation mAbs and combination strategies in early clinical evaluation. Ongoing molecular surveillance for resistance-associated mutations across all three agents will be essential to safeguard the long-term durability of current prophylactic strategies [13,39,42,43].

## Figures and Tables

**Table 1:** Comparative profile of palivizumab, nirsevimab and clesrovimab for RSV prevention [12,15,17,20,21,34].

Agent	Palivizumab	Nirsevimab	Clesrovimab
**Mechanism**	Passive immunoprophylaxis; intramuscular injection	Passive immunoprophylaxis; intramuscular injection	Passive immunoprophylaxis; intramuscular injection
**Target epitope**	Site II (prefusion and postfusion F protein)	Site Ø (prefusion F protein only)	Site IV (prefusion and postfusion F protein)
**Fc modification**	Unmodified IgG1	YTE triple substitution	YTE triple substitution
**Serum half-life**	~20 days	~71 days	~44 days
**Dosing regimen**	Monthly 15 mg/kg injections during RSV season	Single weight-banded dose (50 mg if <5 kg; 100 mg if ≥5 kg)	Single 105 mg dose; not weight-banded
**Approved population**	High-risk infants only (prematurity ≤ 35 weeks; BPD; hemodynamically significant CHD) in the first season and those remaining at high-risk in the second season (BPD/CHD)	All infants in the first RSV season; high-risk children up to 24 months in the second season	Neonates and infants in the first RSV season
**Neutralization potency**	Reference standard	~10-fold higher than palivizumab	Greater than palivizumab; head-to-head data limited
**Epitope conservation**	Well characterized; shared prefusion/postfusion site II	Highly conserved prefusion-specific site Ø	Binds to highly conserved pre- and postfusion site IV. 99.8% identity across >15,000 RSV A and B sequences
**Resistance risk**	Escape mutations documented in case reports; no evidence of population-level emergence in >25 years of use	Resistant RSV-B variants identified in a small number of breakthrough infections; overall prevalence is low	Data very limited; high epitope conservation suggests low risk
**Regulatory status**	Long established; FDA approval June 1998	FDA approval July 2023; EMA approval October 2022	FDA approval June 2025; EMA approval April 2026

Nirsevimab, clesrovimab and palivizumab require the same storage conditions: refrigeration at 2–8 °C, with instructions not to freeze or shake. Their shelf lives are broadly similar, at 4 years for nirsevimab, 2.5 years for clesrovimab and 3 years for palivizumab. Since these differences are relatively modest and the storage requirements are identical across agents, this will likely minimally influence agent selection. BPD, bronchopulmonary dysplasia; CHD, congenital heart disease; EMA, European Medicines Agency; FDA, Food and Drug Administration; RSV, respiratory syncytial virus; YTE, Met252Tyr/Ser254Thr/Thr256Glu.

**Table 2:** Overview of landmark randomized controlled trials evaluating palivizumab, nirsevimab and clesrovimab [38,46,47,48].

Trial	Agent	Population	Endpoint	Outcome
**IMpact-RSV (1998)** [46]	Palivizumab	Preterm infants ≤ 35 weeks’ gestational age	RSV hospitalization	78% relative risk reduction (95% CI 66–90; *p* < 0.001) vs. placebo
	Palivizumab	Infants with CLD/BPD	RSV hospitalization	39% relative risk reduction (95% CI 20–58; *p* = 0.038) vs. placebo
**Griffin et al.** **(2020)** [47]	Nirsevimab	Preterm infants 29–35 weeks’ gestational age	Medically attended RSV-associated LRTD (primary endpoint)	70.1% relative risk reduction (95% CI 52.3–81.2; *p* < 0.001) vs. placebo
	Nirsevimab	Preterm infants 29–35 weeks’ gestational age	RSV hospitalization (secondary endpoint)	78.4% relative risk reduction (95% CI: 51.9–90.3; *p* < 0.001) vs. placebo
**Hammitt et al. (2022)** [48]	Nirsevimab	Infants ≥ 35 weeks’ gestational age	Medically attended RSV-associated LRTD (primary endpoint)	74.5% relative risk reduction (95% CI: 49.6–87.1; *p* < 0.001) vs. placebo
	Nirsevimab	Infants ≥ 35 weeks’ gestational age	RSV hospitalization (secondary endpoint)	62.1% relative risk reduction (95% CI: −8.6–86.8; *p* = 0.07) vs. placebo
**Zar et al. (2025)** [38]	Clesrovimab	Infants ≥ 29 weeks’ gestational age	Medically attended RSV-associated LRTD (primary endpoint)	60.4% relative risk reduction (95% CI: 44.1–71.9; *p* < 0.001)
	Clesrovimab	Infants ≥ 29 weeks’ gestational age	RSV hospitalization (secondary endpoint)	84.2% relative risk reduction (95% CI: 66.6–92.6; *p* < 0.001)

BPD, bronchopulmonary dysplasia; CI, confidence interval; CLD, chronic lung disease; LRTD, lower respiratory tract disease; RSV, respiratory syncytial virus.

**Table 3:** Comparative safety summary for palivizumab, nirsevimab and clesrovimab [2,12,16,20,21,37].

Feature	Palivizumab	Nirsevimab	Clesrovimab
**Most common adverse events**	Injection site reactions, rash, and fever	Injection site reactions, rash, and fever	Injection site reactions and rash
**Serious adverse event rate**	Comparable to the placebo in trials	Comparable to the placebo in trials	Comparable to the placebo in trials
**Post-marketing safety data**	Extensive; >25 years of surveillance; no new signals identified	Growing real-world dataset; no new signals identified	Not yet available
**Immunogenicity (ADA development)**	Low; no clinical consequence	Low; no impact on PK or efficacy	Low; no impact on PK or efficacy

ADA, anti-drug antibody; PK, pharmacokinetics.

## Data Availability

Not applicable.
